# The impact of community‐based childhood obesity prevention interventions in Australia by socio‐economic position: An individual participant data meta‐analysis

**DOI:** 10.1111/ijpo.70031

**Published:** 2025-06-19

**Authors:** Jane Jacobs, Kathryn Backholer, Steven Allender, Vicki Brown, Liliana Orellana, Rachel Novotny, Luke Wolfenden, Marj Moodie, Melanie Nichols

**Affiliations:** ^1^ Deakin University, Institute for Health Transformation, Global Centre for Preventive Health and Nutrition, School of Health and Social Development, Faculty of Health Geelong Australia; ^2^ Deakin University, Institute for Health Transformation, Deakin Health Economics, School of Health and Social Development, Faculty of Health Geelong Australia; ^3^ Deakin University, Biostatistics Unit, Faculty of Health Geelong Australia; ^4^ Department of Human Nutrition, Food and Animal Sciences University of Hawaii at Manoa, College of Tropical Agriculture and Human Resilience Honolulu Hawaii USA; ^5^ School of Medicine and Public Health, Faculty of Health and Medicine, University of Newcastle Callaghan New South Wales Australia

**Keywords:** childhood obesity, community‐based interventions, dietary intake, physical activity, socio‐economic position

## Abstract

**Introduction:**

Community‐based interventions (CBIs) to prevent childhood obesity show promise in reducing body mass index z‐scores (zBMI). Assessing whether this approach produces equitable outcomes across socio‐economic sub‐groups is typically limited by inadequate sample size. This study aimed to assess the effectiveness of CBIs on zBMI and weight‐related behaviours by socio‐economic position (SEP).

**Methods:**

Individual participant data meta‐analysis using seven Australian childhood obesity prevention CBIs conducted between 2003 and 2022. Trials used consistent intervention approaches, objectively measured height and weight, and self‐ or parent‐reported behaviours, and lasted 2–4 years. Area‐level SEP was estimated using Australian Index of Relative Socio‐economic Advantage and Disadvantage tertiles based on home or school postcodes. Multi‐level linear and logistic models estimated the intervention effect on zBMI and behaviours across SEP levels.

**Results:**

While zBMI scores increased across all groups, the increase was significantly greater in control compared to intervention students (difference: −0.036 [95%CI −0.065, −0.007]), among the 25 346 observations analysed. The effect of CBIs was greater in low compared to high SEP students (intervention effect difference = −0.10 [95%CI −0.18, −0.02]).

**Discussion:**

Obesity prevention CBIs may have greater impacts among children from low SEP areas, potentially contributing to reducing health inequities. Further research is needed to understand barriers to improving weight‐related behaviours across socio‐economic groups.

## 1 INTRODUCTION

In high‐income countries approximately one quarter of children are living with overweight or obesity,^1^ and there is a clear socio‐economic gradient in overweight and obesity prevalence, which increases as socio‐economic position (SEP) decreases.^2^, ^3^, ^4^ While the overall prevalence of childhood overweight and obesity appears to be plateauing in some high‐income countries,^5^ in many cases, this has been shown to conceal widening inequalities between high and low socio‐economic groups.^2^, ^4^, ^6^, ^7^ These inequalities are apparent regardless of whether SEP is measured at an individual level,^6^ by, for example, parent's income or educational achievement, or at area level, using measures such as composite scores derived from neighbourhood demographics.^2^, ^3^, ^8^ Socio‐economic inequalities in overweight and obesity prevalence emerge in early childhood,^7^, ^9^, ^10^ highlighting the importance of intervening in the childhood years to prevent obesity and promote health equity.

Community‐based interventions (CBIs) are multi‐setting and multi‐strategy, often with a focus on structural or systems level changes that are tailored to the community needs.^11^ This approach is recommended by the World Health Organization (WHO)^12^ and has shown promising results in the prevention of childhood obesity, with a meta‐analysis demonstrating a small but significant reduction in BMI z‐score.^13^ By targeting structural factors, CBIs shift the focus away from individual choices and motivations, aiming instead to create an environment that supports and encourages healthier behaviours. A systematic review of community‐based obesity prevention interventions found that strategies aimed at environmental or structural changes were more likely to be effective at preventing weight gain for lower SEP participants, compared to those focused on individual behaviour change.^14^ Another scoping review found that complex interventions, which targeted multiple settings and risk factors, had a lower risk of increasing socio‐economic inequalities compared to those focused on single settings or behaviours.^15^

While CBIs may have socio‐economically equitable impacts, there is a limited amount of empirical research analysing the effect of childhood obesity prevention interventions according to SEP. The available evidence is frequently limited by insufficient statistical power to detect differences between sub‐groups.^16^ The significant heterogeneity in measures of SEP used across and within countries and studies makes comparisons or combined analysis difficult. The application of traditional meta‐analysis techniques to evaluate the impact of other public health interventions by SEP has found similar challenges, particularly due to the large variation in measures of SEP.^17^

Individual participant data meta‐analysis involves the aggregation and analysis of raw individual‐level data from multiple studies.^18^ Unlike traditional meta‐analysis techniques, this approach reduces reliance on published effect sizes and allows for harmonization of measures and the application of a consistent exposure measures across datasets.

This study aimed to investigate whether community‐based childhood obesity prevention interventions in Australia had equitable impacts on weight and weight‐related behaviours across the socio‐economic spectrum, using individual participant data meta‐analysis.

## 2 METHODS

This study was undertaken in line with the Preferred Reporting Items for a Systematic Review and Meta‐analysis of Individual Participant Data (PRISMA‐IPD) Statement.^19^ Ethics approval was granted from Deakin University (DUHREC 2021‐121). This study is part of a larger study titled PRecision Evidence for Childhood obesity prevention InterventionS (PRECIS) which aims to collate and harmonize the individual level data of community‐based obesity prevention interventions targeting children and adolescents.

### 2.1 Study eligibility/inclusion

Studies were included for this analysis if they targeted children and adolescents (aged 0–18 years); used a community‐based intervention approach (e.g., employing multiple interventions, across multiple settings and involving multiple stakeholders); and we were able to obtain the participant level data. For the current study, we analysed harmonized data from six Australian individual level datasets.^20^, ^21^, ^22^, ^23^, ^24^, ^25^ One CBI Healthy Together Victoria (HTV) included both primary (elementary) and secondary school students, with the endpoint measurements taken at different timepoints for each school level (2014–2016 for secondary; 2014–2018 for primary); therefore these were treated separately for the analysis, giving a total of seven trials. Three trials were designed as quasi‐experimental, with longitudinal follow‐up (following the same children over time), and four were cluster randomized controlled trials, with repeat cross‐sectional follow‐up (repeat measures within the same community, but not necessarily the same students; Table 1).

**TABLE 1:** Characteristics of included studies.

Trial (school level)	State	Study design	Data collection design	Baseline‐endpoint year
BAEW (primary)	Victoria	Quasi‐experimental	Longitudinal	2003–2006
IYM (secondary)	Victoria	Quasi‐experimental	Longitudinal	2005–2008
ACT‐IYM (secondary)	Australian Capital Territory	Quasi‐experimental	Longitudinal	2012–2014
HTV—Primary (primary)	Victoria	Cluster randomized controlled trial	Repeat cross‐sectional	2014–2018
HTV—Secondary (secondary)	Victoria	Cluster randomized controlled trial	Repeat cross‐sectional	2014–2016
WHOSTOPS (primary)	Victoria	Cluster randomized controlled trial	Repeat cross‐sectional	2015–2019
RESPOND (primary)	Victoria	Cluster randomized controlled trial	Repeat cross‐sectional	2019–2022

Abbreviations: ACT‐IYM, it's your move (Australian Capital Territory); BAEW, be active eat well; HTV, healthy together Victoria; IYM: it's your move; WHOSTOPS, whole of systems trial of prevention strategies; RESPOND, reflexive evidence and systems interventions to prevention obesity and non‐communicable disease.

### 2.2 Data collection and collation

Each trial included height and weight measurements of children collected according to standard protocols by trained staff, in school or early education settings.^20^, ^21^, ^22^, ^23^, ^24^, ^25^ Clean, de‐identified datasets were provided to the PRECIS study team for harmonization and analysis. The baseline and endpoint measurements of each trial were used for the analysis, and any additional (midpoint) measures were not included in this analysis.

### 2.3 Interventions (independent variables)

The intervention actions for each trial were based around the community‐based approach that was relevant at the time of implementation. These interventions have evolved over time; however, the basic framework of incorporating multiple settings, multiple approaches, and multiple stakeholders, as well as using co‐creation and co‐design to tailor an intervention to the specific community setting, was consistent across all trials. Typically, these approaches aim to make structural or environmental changes that encourage healthy behaviours, rather than focus on individual level behaviour change. Control communities were those not receiving the intervention and were typically in neighbouring communities or other parts of the state.

### 2.4 Outcome measures

The primary outcome was change in body mass index z‐score (zBMI) from baseline to endpoint. The raw height and weight for each participant were used to generate the zBMI, based on the WHO Child Growth Reference.^26^

Six behavioural outcomes were included in the analysis, for which data were available in all seven included trials. Behavioural outcomes were self‐reported by students in all studies apart from one (BAEW) that used parental report. Physical activity outcomes included whether the student used active transport (e.g. walking, cycling) to or from school (yes/no); met screen time guidelines (≤2 h per day) (yes/no) and whether students' physical activity levels were considered high or low. The activity level variable for four trials (HTV primary and secondary, WHOSTOPS, RESPOND) was based on whether they met physical activity guidelines (≥60 min moderate to vigorous physical activity per day) 5 days a week. For BAEW, this was based on how much time the child spent playing outside,^27^ and for IYM and ACT‐IYM, this was based on activity level at school.^21^, ^24^ Dietary outcomes were whether the child met fruit intake guidelines (≥2 serves per day),^21^, ^27^, ^28^, ^29^ consumed one or fewer takeaway meals per week^21^, ^27^, ^28^, ^30^ and consumed less than one sweetened beverage per day.^21^, ^27^, ^28^, ^30^ Questionnaires used for each study are outlined in Table S1.

### 2.5 Socio‐economic position

The Australian Bureau of Statistics (ABS) Index of Socio‐Economic Advantage and Disadvantage (IRSAD) score at the postcode level was used as the SEP indicator. The IRSAD is an area‐level score derived from 25 variables.^31^ A lower score indicates relatively greater disadvantage and lack of advantage, while a high score indicates relatively greater advantage and a lack of disadvantage. The ABS releases updated IRSAD scores with each population census (every 5 years) for a range of geographic classifications, and these are publicly available. For three trials (BAEW, IYM, ACT‐IYM) home postcode was available; this was parent‐reported in one trial (BAEW) and self‐reported in two trials based in secondary schools. School postcode was used for participants in the other four trials (HTV primary and secondary, WHOSTOPS, RESPOND), as they either did not collect home postcode or there were data reliability concerns due to self‐reporting by younger children. As the included trials were conducted at different times over an 18‐year period, the IRSAD scores from the census year that was closest to the baseline measurement year were used for each trial, reflecting the relative economic and social conditions of the community at that timepoint. The Australia‐wide deciles were combined to create three categories: low (Decile 1–3), medium (Decile 4–6) and high (Decile 7–10) SEP. The high SEP category contained four deciles due to lower sample sizes in these deciles.

### 2.6 Statistical analysis

Only students with valid height, weight, age, and binary sex measures (in order to calculate zBMI) and a valid postcode (to calculate SEP) were included in the analyses. Individuals were excluded from the weight‐related behavioural analyses if they had missing data for that behavioural outcome.

A one‐stage meta‐analysis was undertaken. This approach involves analysing all trials simultaneously, while adjusting for clustering within the individual trials.^18^

Multi‐level linear models were fitted to estimate the intervention effect on BMI z‐score. The models included timepoint (baseline/endpoint), condition (intervention/control) and their two‐way interaction to investigate the overall impact of CBIs on BMI z‐score. To investigate the differential impact across SEP groups, the same model was fitted further including SEP and all 2‐way and 3‐way interactions. All models included age, sex, intervention duration, and trial as fixed effects, and school and individual participant identifier as random effects. Participant identifier was included to account for repeated measures in some but not all participants. Marginal estimates (either overall or within each SEP tertile) were calculated for (1) mean zBMI at baseline and endpoint by condition (intervention/control); (2) change in mean BMI z‐score between baseline and endpoint by condition; (3) intervention effect; and (4) for models including SEP, the difference in intervention effect between high SEP (most advantaged, least disadvantaged) and each other tertile.

The effect of CBIs on behavioural outcomes was estimated using multilevel logistic models, including the same fixed and random factors described for the linear models and marginal estimates described above for prevalence of behaviours. A *p* value of <0.05 was considered significant. All data management and analyses were undertaken using Stata SE 18.0.^32^

Sub‐group analysis by school level (primary: aged ~5–12 years, secondary: aged ~13–18 years) was undertaken as previous research has indicated that there may be differential impacts of obesity prevention interventions at different ages and stages of development.^33^, ^34^ The robustness of the main results was tested by conducting a series of ‘leave one out’ sensitivity analyses, to understand the impact of each trial on the results.

## 3 RESULTS

Seven Australian obesity prevention CBIs were included in the analysis. There was a total of 12 642 baseline observations (7493 intervention, 5149 control) and 12 704 endpoint observations (6940 intervention and 5764 control).

At baseline, the mean age of participants in the medium and high SEP groups was significantly different between the intervention and control groups, and there was a lower proportion of females in the high SEP intervention group (Table 2).

**TABLE 2:** Participant baseline characteristics.

	Intervention	Control
*n*		
Low SEP	2487	1648
Med SEP	2355	2252
High SEP	2651	1249
Mean age (SD)		
Low SEP	12.1 (3.2)	12.2 (3.0)
Med SEP	10.6 (2.3)	11.5 (3.1)*
High SEP	13.3 (2.2)	11.0 (3.0)*
Percentage female		
Low SEP	49.1%	48.9%
Med SEP	47.6%	47.8%
High SEP	45.5%	49.5%*

*Note*: *p < 0.05 for two‐sample *t*‐test or proportion test between baseline intervention and control groups within SEP level.

Abbreviation: SEP, Socio‐economic position.

### 3.1 BMI z‐score results

The overall analysis found that from the baseline to the endpoint of the interventions, the mean zBMI increased by 0.036 (0.009, 0.062) in the intervention group, compared to an increase of 0.072 (0.045, 0.099) in the control group. This resulted in a−0.036 (−0.065, −0.007) intervention effect, indicating that the mean zBMI increased to a greater extent in the control compared to the intervention group (*p* = 0.02; Table 3).

**TABLE 3:** Intervention effectiveness on mean BMI z‐score overall, according to SEP and by school level.

	Intervention				Control				Change Endpoint—Baseline		Intervention effect (=difference in change)	Effect modification by SEP	*p*‐value
	Baseline		Endpoint		Baseline		Endpoint		Intervention	Control	Int—control		
	*n*	Beta (95% CI)	*n*	Beta (95% CI)	*n*	Beta (95% CI)	*n*	Beta (95% CI)	Estimate (95%CI)	Estimate (95%CI)	Estimate (95%CI)		
Overall	7493	0.59 (0.55, 0.63)	6940	0.63 (0.59, 0.67)	5149	0.56 (0.52, 0.61)	5764	0.64 (0.60, 0.68)	**0.036 (0.009, 0.062)**	**0.072 (0.045, 0.099)**	**−0.036 (−0.065, −0.007)**		0.015
*By socio‐economic position*													
Low SEP	2487	0.69 (0.63, 0.75)	3179	0.75 (0.69, 0.80)	1648	0.63 (0.57, 0.70)	1625	0.78 (0.71, 0.85)	**0.05 (0.02, 0.09)**	**0.14 (0.10, 0.20)**	**−0.09 (−0.15, −0.04)**	**−0.10 (−0.18, −0.02)**	0.011
Med SEP	2355	0.55 (0.49, 0.62)	1658	0.60 (0.53, 0.67)	2252	0.55 (0.48, 0.60)	3136	0.62 (0.56, 0.68)	0.04 (−0.01, 0.09)	**0.07 (0.04, 0.11)**	−0.03 (−0.09, 0.03)	−0.04 (−0.12, 0.04)	0.36
High SEP	2651	0.51 (0.44, 0.58)	2103	0.53 (0.46, 0.59)	1249	0.47 (0.39, 0.55)	1003	0.48 (0.40, 0.56)	0.02 (−0.02, 0.05)	0.01 (−0.03, 0.05)	0.01 (−0.04, 0.06)	ref	
*Primary schools only*													
Low SEP	1334	0.75 (0.68, 0.83)	1333	0.82 (0.74, 0.89)	993	0.69 (0.60, 0.77)	931	0.87 (0.78, 0.96)	**0.06 (0.01, 0.12)**	**0.18 (0.11, 0.26)**	**−0.12 (−0.20, −0.04)**	−0.07 (−0.24, 0.09)	0.39
Med SEP	2100	0.66 (0.59, 0.73)	1501	0.70 (0.63, 0.77)	1379	0.65 (0.57, 0.73)	1763	0.72 (0.64, 0.79)	0.04 (−0.02, 0.09)	**0.07 (0.02, 0.11)**	−0.03 (−0.10, 0.04)	0.02 (−0.14, 0.17)	0.81
High SEP	583	0.70 (0.59, 0.82)	424	0.63 (0.51, 0.76)	824	0.56 (0.46, 0.65)	801	0.54 (0.44, 0.63)	−0.07 (−0.20, −0.06)	−0.02 (−0.08, 0.04)	−0.05 (−0.19, 0.09)	*ref*	
*Secondary schools only*													
Low SEP	1160	0.58 (0.49, 0.66)	1852	0.63 (0.55, 0.71)	717	0.57 (0.47, 0.68)	696	0.70 (0.60, 0.80)	0.05 (−0.00, 0.11)	**0.13 (0.06, 0.20)**	−0.07 (−0.15, 0.01)	−0.02 (−0.13, 0.09)	0.69
Med SEP	256	0.33 (0.14, 0.50)	157	0.48 (0.29, 0.67)	876	0.40 (0.31, 0.49)	1384	0.50 (0.41, 0.58)	0.15 (−0.02, 0.33)	**0.10 (0.04, 0.15)**	0.05 (−0.12, 0.23)	0.10 (−0.09, 0.30)	0.29
High SEP	2077	0.37 (0.30, 0.45)	1695	0.42 (0.34, 0.50)	426	0.44 (0.28, 0.59)	212	0.53 (0.37, 0.69)	**0.04 (0.00, 0.09)**	**0.09 (0.02, 0.17)**	−0.05 (−0.12, 0.02)	*ref*	

*Note*: Results of multi‐level linear regressions estimating change in mean BMI z‐score for all participants, stratified by SEP, and according to school level (primary or secondary). Regressions adjusted for age, sex, trial and trial duration, and clustering at individual and school level. Boldface: *p* < 0.05.

When stratified by SEP, there was a significant intervention effect in the low SEP group, whereby the mean zBMI of the control group increased by 0.14 (95% CI 0.10, 0.20) compared to an increase of 0.05 (95%CI 0.02, 0.09) in the intervention group, resulting in a −0.09 (95%CI −0.15, −0.04) mean difference (*p* = 0.02). There was no significant intervention effect on mean zBMI in the medium or high SEP groups. When compared to the effect in the high SEP group, there was a significantly greater intervention effect in the low SEP group (difference in mean BMI z‐score change = −0.10 (−0.18, −0.02); *p* = 0.01).

Stratifying trials into those where outcomes were measured in primary school‐aged students and secondary school‐aged students found that the significant result persisted in students living or attending schools in low SEP areas for primary school students, with an intervention effect on mean zBMI of −0.12 (95% CI −0.20, −0.04). However, this was not significantly different to the change in the high SEP group. There was no significant intervention effect in the secondary students; however, there was a similar pattern of increased mean zBMI within both the intervention and control groups.

Testing the sensitivity of the results using the ‘leave one out’ analyses, a similar pattern in the increase in zBMI in both control and intervention groups persisted, particularly in the low SEP group. However, when leaving out BAEW, the intervention effect was no longer significant in the low SEP group, and the difference between high and low SEP groups was also non‐significant. (Table S3).

### 3.2 Behavioural results

**FIGURE 1:**
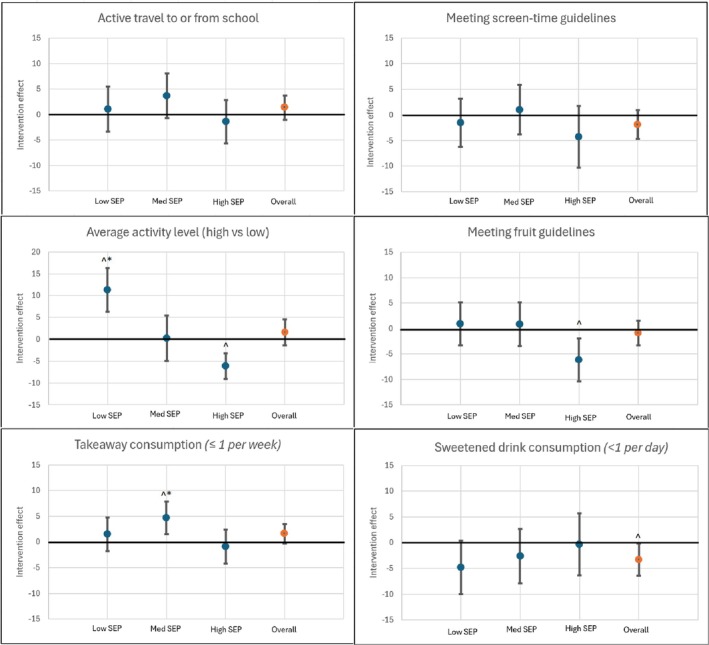
Intervention effect on weight‐related behaviours. Intervention effect (change in proportion of participants meeting recommendations/guidelines between baseline and endpoint) on weight‐related behaviours from mixed effect logistic regression models. Negative results indicate greater change in control group; positive results indicate greater change in intervention group. ^*p* < 0.05 for intervention effect. *p < 0.05 for difference in intervention effect with high SEP.

There was a positive intervention effect on activity level for low SEP students, with an increase in the proportion of students that were highly active in the intervention group relative to the control group (difference = 11.3 [95%CI 6.3, 16.4]) (Figure 1). High SEP students' activity levels in the control group increased to a greater degree than those in the intervention group. This resulted in a 17.4 percentage point (95% CI 9.8, 25.1) difference in the intervention effect in low compared to high SEP areas, on the proportion of students being highly active. There was an increase in the proportion of high SEP control students meeting fruit consumption guidelines (5.7 [95%CI: 2.0, 9.4]), resulting in a significant difference in the intervention effect on high compared to low (difference: 7.0 [95%CI 0.8, 13.2]) and medium (difference: 6.9 [95%CI 0.6, 13.2]) SEP students. A positive intervention effect was observed for the change in the proportion of students consuming one or less takeaway meals per week (4.7 [95%CI 1.5, 7.8]) for those living or going to school in medium SEP areas, which was significantly higher than the intervention effect for high SEP students (difference: 5.6 [95%CI 0.8, 10.4]).

Overall, control students reduced their sugar sweetened beverage intake, with the proportion of those consuming less than one sugar sweetened beverage increasing between the baseline and endpoint (3.0, 95%CI 0.7, 5.3), resulting in a negative intervention effect of −3.3 percentage points, (95% CI −6.4, −0.2). There were no significant results for active transport or meeting screen time guidelines overall, or by SEP (Table S4).

## 4 DISCUSSION

Using pooled individual level data from seven Australian community‐based obesity prevention trials, we demonstrate that these interventions can be effective at slowing weight gain, with greater benefits among children who live, or go to school, in lower SEP areas. Specifically, we found that while zBMI increased for both the pooled intervention and comparison groups, the increase was higher in the comparison group, indicating the interventions were effective in slowing the increasing BMI trajectory. This finding was greater for primary school children with a lower SEP compared to primary school children with a higher SEP (where no intervention effect as observed), with no differences in intervention effects between secondary school children of a low or high SEP. However, testing the robustness of our results with a ‘leave one out’ analysis indicates that many of the results may have been driven by one of the earlier studies in our analysis, particularly those observed in the high SEP group.

Our findings align with existing evidence that suggests that CBIs may be effective at reducing socioeconomic inequities in overweight and obesity.^14^, ^15^ While prior studies have generally been underpowered to detect sub‐group differences, systematic reviews and syntheses of existing literature have shown that CBIs that include structural changes to food and physical activity environments are associated with positive intervention effects for children with a low SEP.^15^, ^35^ Our study is the first to empirically confirm and extend this finding by showing that CBI intervention effects not only benefit low SEP children but to a greater degree than high SEP children. This means that CBIs may be a promising strategy to narrow the unacceptable inequities in obesity prevalence between those with a higher and lower SEP.^4^, ^6^, ^7^

Our finding that obesity prevention CBIs did not have a significant impact on zBMI for children from a high SEP diverges from some prior research.^13^ One potential explanation for this is that higher SEP groups may have less potential for zBMI improvement; our results indicate that these students with a higher SEP had lower zBMI at baseline. Nevertheless, zBMI increased for all SEP sub‐groups across the intervention period, indicating a need to prevent excess weight gain for all children.

Our overall results are in line with, although smaller than, a systematic review of ‘whole of community’ childhood obesity prevention interventions, which found a significant intervention effect on BMIz of −0.09 (95%CI −0.16, −0.02).^13^ While we found a lower estimate, significant health benefits and health care cost savings have been found at the population level with only small changes in zBMI.^36^, ^37^ For example, using the Assessing Cost‐Effectiveness (ACE) model, Ananthapavan et al.^38^ found that an intervention effect on BMIz of −0.07 (95%CI −0.13, −0.01), would result in $452 M in healthcare cost savings and 51 792 health adjusted life years (HALYs) gained.

Our results for primary school children contrast with a recent large Cochrane systematic review of 172 interventions aimed at preventing obesity among children aged 5 to 11 years old.^33^ The Cochrane review concluded that there was limited evidence supporting the effectiveness of obesity prevention, particularly within school settings. Importantly, the Cochrane review synthesized trial outcomes from a wide range of published studies, relying solely on published results. Our study, on the other hand, pooled individual‐level data from trials conducted in the same country, with similar methods. The Cochrane review also focussed on overall effects, with authors reporting that only 8% (*n* = 14) of studies had reported the impact of socio‐economic status on the effectiveness of the intervention.^33^ However, these results were not synthesized due to heterogeneity in SEP measures, which the review identified as a key limitation. This lack of differential analysis may mask important sub‐group differences, which have been explored in our study.

Our findings, showing no significant impact for obesity prevention CBIs for any secondary school SEP groups, align with another recent systematic review examining the effects of obesity prevention among secondary students (age 12–19 years), which also found inconclusive evidence for the effectiveness of obesity prevention interventions among adolescents.^34^ This lack of significant results in the secondary students may also have contributed to a lack of significant results in the high SEP group. Many of the secondary students in the intervention groups were living or going to school in high SEP areas, and it is more difficult to have an impact on behaviours and weight status in older students.^34^ This discrepancy in impact between primary and secondary school‐aged children and adolescents points to the importance of early intervention on weight gain, and establishing healthy weight‐related behaviours in early childhood, as it appears that the impact of interventions may be limited as students move into the adolescent years.

Our behavioural results indicated a positive intervention effect on the proportion of low SEP students being highly active, while the proportion of high SEP control students meeting this recommendation increased and the high SEP intervention stayed consistent, resulting in a significant negative intervention effect for these students. A US obesity prevention CBI, the Healthy Living Cambridge Kids study, analysed outcomes according to SEP and found equal improvement in fitness level across SEP,^39^ although in contrast with our study, an individual level measure of SEP was used (eligibility for free school lunches). Similar to the activity level results, the negative intervention effect on meeting fruit guidelines for high SEP students was primarily a result of an increase in consumption for control communities, while consumption in intervention communities remained relatively consistent. The most recent Australian Health Survey found that children from higher SEP areas consume more fruit,^40^ which is reflected in our data, which showed that a greater proportion of children and adolescents in the high SEP groups met fruit guidelines at baseline.

## 5 STRENGTHS

The data used in this study came from multiple high‐quality long‐term community‐based obesity prevention interventions. Homogeneity in study design has been recognized as a critical element required for pooled analysis.^41^ The trials included in our analysis were conducted with highly specified protocols and the same primary outcome measure of zBMI collected by trained staff. The trials were also highly specified, and this analysis was strengthened by using data from trials with strong overlap in terms of intervention approach and ethos. Being able to apply a consistent measure of SEP across trials also enhanced comparability between trials.

This study included sensitivity analysis to explore whether individual trials were having differential impact on analyses outcomes when included in the pooled analysis. This allowed us to see that the Be Active Eat Well trial^22^ was a key driver of the observed effects that favoured intervention. The results remained in the same direction with removal of this trial but the magnitude of effect was greatly reduced and the result did not reach significance. This was the earliest trial included in our study and it is possible that there were more gains to be made in earlier iterations of obesity prevention CBIs, before the implementation of more widespread policies and practices, especially in schools, to improve healthy behaviours.^42^, ^43^

## 6 LIMITATIONS

While intervention and evaluation approaches were similar across all included trials, the development of intervention strategies within trials varied according to trial context and the aims of each community, and there was development in knowledge of intervention approaches and theory over time. The core implementation principle, of building community capacity to adapt and apply the existing evidence to the local context, meant that the specific strategies in each CBI differed from those in other communities and trials included in the analysis. Due to inclusion criteria of requiring record‐level data, not all obesity prevention CBIs conducted in Australia during this time were included. However, based on a recent systematic review that examined the range of outcome measures used in obesity prevention CBIs, we did include six out of seven relevant Australian studies.^41^

Each trial collected self‐reported data for child behaviours, with the exception of one trial that used parental report (BAEW). The use of self‐report is known to be less reliable than observed or objectively measured data, being subject to recall and social‐desirability biases and being reliant on student comprehension of questions.^44^ However, each study reported that the questionnaires used were validated in the target population. While the use of standard socio‐economic measures between the trials is a strength, the analysis relies on the measure collected at an area rather than an individual level. As such, it reflects the relative advantage and deprivation of the area in which the child lives or goes to school, rather than the circumstances of the individual child. We were not able to include individual level SEP indicators as the demographic information was reported by children and adolescents, and it would not have been feasible to ask information such as parental education or income (which are common individual or family level SEP indicators). Furthermore, we relied on school postcode for four of the studies due to a lack of accurate home postcode reporting in these studies. However, in many of these rural communities, there is little variation between the school and home postcode. Research indicates that both individual and area level SEP measures result in consistent socio‐economic gradients in health outcomes, particularly chronic disease,^45^, ^46^ though each may exert influence in different ways. Further, with the focus of the included trials often being environmental change at the community level, an area level measure of SEP may be most appropriate. While the trials were each of different durations, the adjustment for length of intervention allowed for the impact of time exposed to the intervention condition.

A further limitation is that ages and SEP levels were not evenly distributed between trials and between trial arms at baseline. Such discrepancies may have contributed to our results. Our harmonization process, which involved standardizing diverse questions and responses into meaningful binary outcomes, may have reduced the specificity of individual responses and the granularity of some intended study outcomes. Additionally, we were not able to include meeting vegetable guidelines, a key dietary variable, as we did not have comparable and consistent data across the studies in relation to vegetable intake.

## 7 IMPLICATIONS FOR POLICY AND PRACTICE

The differential effects of obesity prevention CBIs by SEP that we observed in our study suggest that these types of interventions may contribute to reducing socioeconomic inequities in obesity prevalence. However, there was heterogeneity in this effect across trials, and the lack of impact in medium and high SEP groups, where overweight and obesity remain a public health concern, raises questions about the relevance of such interventions as a universal approach to obesity prevention. Future CBIs should consider the principles of proportionate universalism, where an intervention is universal in nature but implemented with an intensity and resources that are proportionate to the level of need.^47^ Greater standardization of measures collected in trial evaluations would allow for more accurate synthesis of results across future interventions and investigation of sub‐group effects, as well as enhancing external validity of results.^41^

## 8 CONCLUSIONS

The results of this individual participant data meta‐analysis indicate that community‐based interventions for obesity prevention may be more effective in slowing weight gain among children living or attending schools in areas of lower SEP, compared to those of a higher SEP. However, the findings for behavioural outcomes are less clear. Further research is needed to explore the driving factors of successful interventions, and to understand how these factors might differ across SEP levels. This would provide valuable insights for the future design and implementation of effective and equitable CBIs.

## AUTHOR CONTRIBUTIONS

Melanie Nichols, Jane Jacobs conceptualized the study. Melanie Nichols, Jane Jacobs, Liliana Orellana, Kathryn Backholer, and Steven Allender designed the study methodology. Melanie Nichols and Steven Allender obtained the raw data. Jane Jacobs and Melanie Nichols collated the data, and Jane Jacobs conducted the analysis with assistance from Melanie Nichols, Liliana Orellana, and Kathryn Backholer. Jane Jacobs, Kathryn Backholer, Melanie Nichols, and Steven Allender wrote the original manuscript draft, with input from Luke Wolfenden, Vicki Brown, Rachel Novotny, and Marj Moodie. All authors contributed to reviewing and editing the manuscript and approved the final version prior to submission.

## FUNDING INFORMATION

This study was supported by an NHMRC Ideas Grant (GNT2002334). RN is supported by the following grants: USDA 2021‐68012‐35899, NIH 1P20GM139753‐01A1, NIH T32DK137523. KB is supported by a Fellowship [106716] from the National Heart Foundation of Australia. The contents of this publication are solely the responsibility of the authors and do not reflect the views of the funding bodies.

## CONFLICT OF INTEREST STATEMENT

The authors declare no conflict of interest.

## Supporting information


**DATA S1.** Supporting Information.
