# Supplementary material for: The impact of community‐based childhood obesity prevention interventions in Australia by socio‐economic position: An individual participant data meta‐analysis
Source: Pediatr Obes. 2025 Jun 19;20(10):e70031. doi: 10.1111/ijpo.70031 (PMC12414578; doi:10.1111/ijpo.70031)
Supplement: Supplementary file 1 — DATA S1. Supporting Information. [file IJPO-20-e70031-s001.pdf]

Title: The impact of community-based childhood obesity prevention interventions in Australia by socio-economic position: An individual participant data meta-analysis.

Authors: Jane Jacobs[1] (corresponding author), Kathryn Backholer[1], Steven Allender[1], Vicki Brown[1,2], Liliana Orellana[3], Rachel Novotny[4], Luke Wolfenden[5], Marj Moodie[1,2], Melanie Nichols[1]

[1] Deakin University, Institute for Health Transformation, Global Centre for Preventive Health and Nutrition, School of Health and Social Development, Faculty of Health, Geelong, Australia,

[2] Deakin University, Institute for Health Transformation, Deakin Health Economics, School of Health and Social Development, Faculty of Health, Geelong, Australia,

[3] Deakin University, Biostatistics Unit, Faculty of Health, Geelong, Australia,

[4] University of Hawaii at Manoa, College of Tropical Agriculture and Human Resilience, Department of Human Nutrition, Food and Animal Sciences

[5] School of Medicine and Public Health, Faculty of Health and Medicine, University of Newcastle, Callaghan, NSW, 2308, Australia

Corresponding Author: Jane Jacobs , Deakin University. 1 Gheringhap st, Geelong, Victoria, Australia, 3220. Email: jane.jacobs@deakin.edu.au

Supplementary Table 1: Distribution of participants across SEP groups used in the analysis, within trials

	Baseline participants: Intervention (n)			Baseline participants: Control (n)			Endpoint participants: Intervention (n)			Endpoint Participants: Control (n)		
**Trial (school level)**	**Low SEP**	**Med SEP**	**High SEP**	**Low SEP**	**Med SEP**	**High SEP**	**Low SEP**	**Med SEP**	**High SEP**	**Low SEP**	**Med SEP**	**High SEP**
BAEW (primary)	563	246	3	154	491	308	563	246	3	154	491	308
IYM (secondary)	285	21	942	190	479	59	285	21	942	190	479	59
ACT - IYM (secondary)	0	0	480	1	0	143	0	0	480	1	0	143
HTV – Primary (primary)	512	843	297	376	248	143	463	437	215	405	386	92
HTV – Secondary (secondary)	868	234	646	524	394	223	1561	136	257	503	894	0
WHOSTOPS (primary)	201	626	151	69	528	218	226	542	104	110	813	307
RESPOND (primary)	58	385	132	334	112	155	80	276	102	262	73	94

BAEW: Be Active Eat Well; IYM: It’s Your Move; IYM – ACT: It’s Your Move (Australian Capital Territory); HTV: Healthy Together Victoria; WHOSTOPS: Whole of Systems Trial of Prevention Strategies; RESPOND: Reflexive Evidence and Systems interventions to Prevention Obesity and Non-communicable Disease

Supplementary Table 2: Questionnaires and survey tools used in each included trial

	BAEW	IYM-ACT	IYM	HTV primary/secondary	WHOSTOPS	RESPOND
**Active travel**	Eating Patterns & Physical Activity in Kinder/primary school children	Adolescent Behaviours, Attitudes and Knowledge Questionnaire	Adolescent Behaviours, Attitudes and Knowledge Questionnaire	School Health Action, Planning and Evaluation System questionnaire (Wong et al)	Project developed question regarding mode of travel to/from school	Project developed question regarding mode of travel to/from school
**Activity level**	Eating Patterns & Physical Activity in Kinder/primary school children	Adolescent Behaviours, Attitudes and Knowledge Questionnaire	Adolescent Behaviours, Attitudes and Knowledge Questionnaire	Canadian Core Indicators and Measures of Youth Health – Physical Activity and Sedentary Behaviour Module questionnaire (Card et al)	Canadian Core Indicators and Measures of Youth Health – Physical Activity and Sedentary Behaviour Module questionnaire (Card et al)	Canadian Core Indicators and Measures of Youth Health – Physical Activity and Sedentary Behaviour Module questionnaire (Card et al)
**Screen Time**	Eating Patterns & Physical Activity in Kinder/primary school children	Adolescent Behaviours, Attitudes and Knowledge Questionnaire	Adolescent Behaviours, Attitudes and Knowledge Questionnaire	Canadian Core Indicators and Measures of Youth Health – Physical Activity and Sedentary Behaviour Module questionnaire (Card et al)	Canadian Core Indicators and Measures of Youth Health – Physical Activity and Sedentary Behaviour Module questionnaire (Card et al)	Canadian Core Indicators and Measures of Youth Health – Physical Activity and Sedentary Behaviour Module questionnaire (Card et al)
**Fruit consumption**	Eating Patterns & Physical Activity in Kinder/primary school children	Adolescent Behaviours, Attitudes and Knowledge Questionnaire	Adolescent Behaviours, Attitudes and Knowledge Questionnaire	Simple Dietary Questionnaire (Parletta et al)	Simple Dietary Questionnaire (Parletta et al)	Child Nutrition Questionnaire (Wilson et al)
**Takeaway consumption**	Eating Patterns & Physical Activity in Kinder/primary school children	Adolescent Behaviours, Attitudes and Knowledge Questionnaire	Adolescent Behaviours, Attitudes and Knowledge Questionnaire	Simple Dietary Questionnaire (Parletta et al)	Simple Dietary Questionnaire (Parletta et al)	Food, Health and Choices Questionnaire (Gray et al)
**SSB consumption**	Eating Patterns & Physical Activity in Kinder/primary school children	Adolescent Behaviours, Attitudes and Knowledge Questionnaire	Adolescent Behaviours, Attitudes and Knowledge Questionnaire	Simple Dietary Questionnaire (Parletta et al)	Simple Dietary Questionnaire (Parletta et al)	Food, Health and Choices Questionnaire (Gray et al)

BAEW: Be Active Eat Well; IYM: It's Your Move; IYM – ACT: It's Your Move (Australian Capital Territory); HTV: Healthy Together Victoria; WHOSTOPS: Whole of Systems Trial of Prevention Strategies; RESPOND: Reflexive Evidence and Systems interventions to Prevention Obesity and Non-communicable Disease

Supplementary Table 3: Leave one out analysis

	Intervention				Control				Change Endpoint - Baseline		Intervention effect (= difference in change)	Effect modification by SEP^	P value
	Baseline		Endpoint		Baseline		Endpoint		Intervention	Control	Int - control		
	n	beta (95% CI)	n	beta (95% CI)	n	beta (95% CI)	n	beta (95% CI)	estimate (95%CI)	estimate (95%CI)	estimate (95%CI)		
Leave out BAEW													
Low SEP	1924	0.66(0.59, 0.73)	2615	0.73(0.66, 0.79)	1496	0.63(0.55, 0.70)	1471	0.73(0.65, 0.80)	**0.06(0.01,0.11)**	**0.10(0.04, 0.16)**	-0.04(-0.11, 0.04)	0.01(-0.09, 0.11)	0.91
Med SEP	2109	0.52(0.45, 0.59)	1412	0.58(0.50, 0.66)	1761	0.50(0.43, 0.57)	2645	0.58(0.51, 0.64)	0.06(-0.02,0.13)	**0.07(0.03,0.12)**	-0.02(-0.10, 0.06)	0.03(-0.08, 0.13)	0.65
High SEP	2648	0.48(0.42, 0.55)	2100	0.51(0.44, 0.57)	941	0.39(0.29, 0.48)	695	0.45(0.36, 0.55)	0.02(-0.02,0.06)	**0.06(0.00, 0.13)**	-0.04(-0.11, 0.02)	ref	
Leave out IYM													
Low SEP	2202	0.70(0.64, 0.77)	2894	0.75(0.69, 0.81)	1458	0.62(0.54, 0.70)	1435	0.80(0.72, 0.87)	**0.05(0.00, 0.09)**	**0.18(0.11, 0.24)**	**-0.13(-0.20, -0.06)**	**-0.14(-0.24, -0.04)**	0.01
Med SEP	2334	0.57(0.50, 0.64)	1637	0.60(0.53, 0.67)	1773	0.59(0.52, 0.66)	2657	0.64(0.57, 0.71)	0.03(-0.03, 0.08)	**0.05(0.01, 0.10)**	-0.03(-0.09, 0.04)	-0.04(-0.13, 0.06)	0.42
High SEP	1709	0.55(0.47, 0.63)	1161	0.54(0.46, 0.63)	1190	0.50(0.41, 0.58)	944	0.48(0.39, 0.56)	-0.01(-0.07, 0.05)	-0.02(-0.07, 0.03)	0.01(-0.06, 0.08)	ref	
Leave out IYM – ACT													
Low SEP	2487	0.70(0.64, 0.76)	3179	0.75(0.70, 0.81)	1647	0.64(0.57, 0.71)	1624	0.79(0.72, 0.85)	**0.05(0.02, 0.09)**	**0.15(0.10, 0.20)**	**-0.09(-0.15, -0.04)**	**-0.10(-0.19, -0.02)**	0.02
Med SEP	2355	0.56(0.49, 0.62)	1658	0.60(0.54, 0.67)	2252	0.55(0.49, 0.61)	3136	0.62(0.56, 0.68)	0.04(-0.01, 0.09)	**0.07(0.04, 0.11)**	-0.03(-0.09, 0.03)	-0.04(-0.13, 0.05)	0.40
High SEP	2171	0.54(0.47, 0.61)	1623	0.54(0.47, 0.61)	1106	0.47(0.38, 0.55)	860	0.46(0.37, 0.55)	0.00(-0.04, 0.05)	-0.01(-0.06, 0.05)	0.01(-0.05, 0.07)	ref	
Leave out HTV - primary													
Low SEP	1975	0.69(0.62, 0.75)	2716	0.73(0.67, 0.79)	1272	0.62(0.54, 0.70)	1220	0.77(0.69, 0.85)	**0.05(0.01, 0.09)**	**0.15(0.11, 0.21)**	**-0.11(-0.17, -0.05)**	**-0.12(-0.20, -0.04)**	0.003
Med SEP	1512	0.53(0.46, 0.61)	1221	0.57(0.50, 0.65)	2004	0.52(0.46, 0.58)	2750	0.59(0.53, 0.65)	0.04(-0.01, 0.10)	**0.07(0.04, 0.11)**	-0.03(-0.08, 0.03)	-0.04(-0.12, 0.04)	0.36
High SEP	2354	0.48(0.41, 0.55)	1888	0.50(0.43, 0.57)	1106	0.48(0.39, 0.57)	911	0.49(0.40, 0.57)	0.02(-0.02, 0.05)	0.01(-0.04, 0.05)	0.01(-0.04, 0.07)	Ref	
Leave out HTV – secondary													
Low SEP	1736	0.72(0.65, 0.79)	1627	0.78(0.71, 0.85)	1185	0.68(0.60, 0.76)	1124	0.82(0.74, 0.90)	**0.06(0.02, 0.10)**	**0.14(0.09, 0.20)**	**-0.09(-0.15, -0.02)**	**-0.09(-0.17, -0.01)**	0.03
Med SEP	2165	0.61(0.55, 0.68)	1522	0.66(0.59, 0.73)	1952	0.58(0.51, 0.64)	2254	0.65(0.59, 0.72)	0.05(-0.00, 0.10)	**0.08(0.04, 0.11)**	-0.03(-0.09, 0.03)	-0.03(-0.11, 0.05)	0.45
High SEP	2016	0.56(0.49, 0.63)	1862	0.57(0.50, 0.65)	1076	0.52(0.43, 0.60)	1013	0.53(0.45, 0.61)	0.02(-0.02, 0.06)	0.01(-0.03, 0.06)	0.00(-0.05, 0.06)	ref	
Leave out WHOSTOPS													
Low SEP	2286	0.67(0.61, 0.74)	2953	0.73(0.67, 0.79)	1579	0.64(0.56, 0.71)	1515	0.79(0.71, 0.86)	**0.05(0.02, 0.09)**	**0.15(0.10, 0.20)**	**-0.10(-0.16, -0.04)**	**-0.10(-0.19, -0.02)**	0.01
Med SEP	1729	0.52(0.44, 0.60)	1116	0.55(0.47, 0.63)	1724	0.54(0.47, 0.61)	2323	0.62(0.55, 0.68)	0.03(-0.03, 0.09)	**0.07(0.04, 0.11)**	-0.04(-0.11, 0.02)	-0.05(-0.14, 0.04)	0.25
High SEP	2500	0.48(0.40, 0.55)	1999	0.49(0.42, 0.57)	1031	0.44(0.35, 0.54)	696	0.46(0.36, 0.55)	0.02(-0.02, 0.05)	0.01(-0.04, 0.06)	0.01(-0.05, 0.06)	ref	
Leave out RESPOND													
Low SEP	2429	0.68(0.62, 0.74)	3099	0.74(0.69, 0.80)	1314	0.63(0.56, 0.70)	1363	0.79(0.72, 0.86)	**0.06(0.02, 0.10)**	**0.16(0.11, 0.21)**	**-0.10(-0.16, -0.04)**	**-0.11(-0.19, -0.03)**	0.01
Med SEP	1970	0.56(0.49, 0.63)	1382	0.62(0.55, 0.69)	2140	0.54(0.48, 0.61)	3063	0.63(0.57, 0.68)	**0.06(0.01, 0.11)**	**0.08(0.05, 0.12)**	-0.02(-0.08, 0.04)	-0.04(-0.12, 0.04)	0.37
High SEP	2519	0.51(0.45, 0.58)	2001	0.54(0.47, 0.61)	1094	0.50(0.41, 0.58)	909	0.51(0.43, 0.60)	0.03(-0.01, 0.06)	0.01(-0.03, 0.06)	0.01(-0.04, 0.07)	ref	

Results of mixed-effects logistic regressions estimating change in proportion meeting the behavioural guidelines/recommendations, leaving out one study each time. Regressions adjusted for age, sex, trial and trial duration, and clustering at individual and school level. **Boldface**: p<0.05

*did not adjust for individual repeat measures due to non-convergence

Supplementary Table 4: Intervention effectiveness on behavioural outcomes, by SEP

	Intervention				Control				Change Endpoint - Baseline		Intervention effect (= difference in change)	Effect modification by SEP^	P value
	Baseline		Endpoint		Baseline		Endpoint		Intervention	Control	Int - control		
	n	proportion (95% CI)	n	proportion (95% CI)	n	proportion (95% CI)	n	proportion (95% CI)	proportion (95%CI)	proportion (95%CI)	proportion (95%CI)	proportion (95%CI)	
Active transport to or from school													
Low SEP	2077	40.3(35.5, 45.0)	2899	40.5(35.9, 45.0)	1380	37.0(31.8, 42.3)	1419	36.1(31.3, 41.2)	0.2(-2.6, 3.0)	-0.9(-4.3, 2.5)	1.1(-3.2, 5.5)	2.5(-3.7, 8.7)	0.43
Med SEP	1849	23.5(19.1, 27.8)	1337	25.2(20.7, 29.7)	1897	38.9(33.9, 43.8)	2690	36.8(32.1, 41.6)	1.7(-1.6, 5.1)	-2.0(-4.8, 0.8)	3.7(-0.6, 8.1)	5.1(-1.1,11.3)	0.11
High SEP	2392	27.9(23.4, 32.4)	2009	27.9(23.4, 32.4)	1048	30.6(25.1, 36.2)	845	32.0(26.4, 37.6)	0.0(-2.4, 2.4)	1.4(-2.4, 5.1)	-1.4(-5.7, 3.0)	ref	
Activity level (high vs low)													
Low SEP	2059	34.5(30.9, 38.1)	2708	43.0(39.4, 46.6)	1374	38.7(34.3, 43.0)	1335	35.8(31.6, 40.0)	**8.5(5.3, 11.7)**	-2.8(-6.8, 1.2)	**11.3(6.3, 16.4)**	**17.4(9.8, 25.1)**	<0.01
Med SEP	1757	37.5(33.4, 41.7)	1288	43.6(39.1, 48.2)	1860	36.3(32.4, 40.4)	2574	42.2(38.4, 46.1)	**6.1(1.9, 10.3)**	**5.9(2.6, 9.2)**	0.2(-5.1, 5.4)	6.3(-1.6, 14.2)	0.11
High SEP	2353	36.9(32.8, 41.0)	1967	37.1(32.9, 41.3)	1078	40.4(35.4, 45.4)	833	46.7(41.5, 52.0)	0.2(-3.1, 3.5)	**6.3(1.5, 11.2)**	**-6.1(-11.9, -0.4)**	ref	
Meeting screen-time guidelines (Two hours or less per day)													
Low SEP	2020	65.9(62.4, 69.4)	2723	66.4(63.1, 69.6)	1382	65.9(61.8, 69.9)	1357	67.9(64.0, 71.7)	0.5(-2.5, 3.8)	2.0(-1.6, 5.6)	-1.5(-6.2, 3.1)	2.8 (-4.9, 10.3)	0.48
Med SEP	1767	70.8(67.0, 74.6)	1297	71.7(67.8, 75.6)	1882	67.5(63.8, 71.2)	2584	67.4(64.0, 70.9)	0.9(-2.9, 4.7)	-0.1(-3.1, 3.0)	1.0(-3.8, 5.8)	5.2(-2.5, 13.0)	0.19
High SEP	2333	66.5(62.7, 70.3)	1830	62.5(58.4, 66.5)	961	66.4(61.4, 71.5)	780	66.7(61.7, 71.6)	**-4.0(-7.2, -0.9)**	0.2(-5.0, 5.4)	-4.3(-10.3, 1.7)	ref	
Meeting fruit guidelines (Two or more per day)													
Low SEP	2263	65.3(62.5, 68.5)	2872	67.5(64.9, 70.2)	1487	65.7(62.3, 69.1)	1426	66.9(63.7, 70.2)	2.1(-0.6, 4.8)	1.2(-2.2, 4.6)	0.9(-3.4, 5.1)	**7.0 (0.8, 13.2)**	0.03
Med SEP	1902	68.8(65.7, 71.9)	1337	69.9(66.6, 73.3)	1969	68.6(65.6, 71.5)	2682	68.9(66.1, 71.6)	1.1(-2.4, 4.6)	0.3(-2.4, 3.0)	0.8(-3.6, 5.1)	**6.9 (0.6, 13.2)**	0.03
High SEP	2515	69.2(66.2, 72.2)	2013	69.0(65.8, 72.1)	1124	70.6(66.9, 74.3)	841	76.3(72.8, 79.8)	-0.5(-3.1, 2.2)	**5.7(2.0, 9.4)**	**-6.2(-10.6, -1.7)**	Ref	
Takeaway consumption (One or less per week)													
Low SEP	2277	85.5(83.2, 87.8)	2928	84.5(82.2, 86.7)	1506	86.8(84.2, 89.4)	1437	84.2(81.4, 87.0)	-1.0(-3.0, 1.0)	-2.5(-5.1, 0.1)	1.5(-1.7, 4.8)	2.5(-2.4, 7.3)	0.32
Med SEP	1934	85.9(83.4, 88.4)	1342	88.4(86.0, 90.9)	1981	88.2(86.0, 90.5)	2711	86.1(83.8, 88.4)	**2.5(0.0, 4.9)**	**-2.2(-4.2,- 0.1)**	**4.7(1.5, 7.8)**	**5.6(0.8, 10.4)**	0.02
High SEP	2526	91.0(89.0, 93.1)	2020	89.4(87.1, 91.7)	1124	88.8(85.9, 91.6)	843	88.0(85.1, 91.0)	-1.7(-3.7, 0.4)	-0.7(-3.7, 2.2)	-0.9(-4.5, 2.6)	ref	
*Sugar sweetened beverage consumption (Less than one per day)**													
Low SEP	2269	68.9(64.9, 72.9)	2794	66.6(62.6, 70.5)	1413	67.8(63.0, 72.5)	1339	70.2(65.8, 74.7)	-2.3(-5.7, 1.1)	2.5(-1.5, 6.5)	-4.8(-10.0, 0.4)	-4.5(-12.4, 3.5)	0.27
Med SEP	1916	68.9(64.5, 73.2)	1332	70.8(66.4, 75.2)	1773	67.4(62.9, 71.8)	2443	71.9(68.0, 75.9)	1.9(-2.1, 6.0)	**4.5(1.1, 8.0)**	-2.6(-7.9, 2.7)	-2.2(-10.3, 5.7)	0.58
High SEP	2113	70.6(66.0, 75.3)	1552	71.1(66.3, 75.8)	1006	69.2(63.9, 74.5)	805	70.0(64.7, 75.1)	0.5(-3.3, 4.2)	0.8(-4.1, 5.6)	-0.3(-6.3, 5.7)	ref	

Results of multi-level linear regressions estimating change in mean BMI z-score, leaving out one study each time. Regressions adjusted for age, sex, trial and trial duration, and clustering at individual and school level.

Boldface: p<0.05
